# Lessons Learned from Governance and Management of Virtual Hospital Initiatives: A Systematic Review

**DOI:** 10.3390/nursrep15120451

**Published:** 2025-12-17

**Authors:** Afrooz Purarjomandlangrudi, Amir Hossein Ghapanchi, Josephine Stevens, Navid Ahmadi Eftekhari, Kirsty Barnes

**Affiliations:** 1 College of Arts, Business, Law, Education and IT, Victoria University, Melbourne 3000, Australia; 2 College of Sport, Health and Engineering, Victoria University, Melbourne 3011, Australia; amir.ghapanchi@vu.edu.au; 3 The Institute for Sustainable Industries and Liveable Cities, Victoria University, Melbourne 3011, Australia; s3985307@student.rmit.edu.au; 4 Western Health, Melbourne 3021, Australia; josephine.stevens@wh.org.au (J.S.); kirsty.barnes@wh.org.au (K.B.)

**Keywords:** hospital in the home, home care services, hospital-based home care, virtual hospital, tele-health, health services administration, organizational innovation, governance, systematic review

## Abstract

**Background:** Hospital In The Home (HITH), also called Hospital at Home or Virtual Hospital, delivers hospital-level care in patients’ homes to enhance outcomes and reduce hospital bed occupancy. Despite widespread implementation, strategic guidance for managing HITH initiatives remains limited. **Methods:** Following PRISMA 2020 guidelines, we conducted a systematic review (protocol not registered) searching ScienceDirect and Scopus (inception to December 2023) using the terms “hospital in the home,” “HITH,” “hospital at home,” “virtual care” AND “lesson,” “management,” “governance.” Peer-reviewed studies reporting lessons learned, best practices, or governance strategies for HITH programs with sufficient implementation detail were included; we excluded studies focusing solely on clinical effectiveness without organizational aspects, conference abstracts, and editorials. Two researchers independently screened records, extracted data, and conducted thematic analysis. Quality assessment used the Mixed Methods Appraisal Tool (MMAT). Sixteen studies (12 high-quality, 3 moderate, 1 low) were included. The studies were moderate overall, based on predominantly observational program evaluations and case studies. **Results:** Forty-two lessons were identified and classified into nine categories: combining care modalities, technology integration, impact on patient outcomes, training and specialized knowledge, care coordination, governance structures, financial sustainability, cross-sector collaboration, and patient selection. These categories fall under four themes: care delivery models; staffing and team dynamics; governance and financial sustainability; and patient selection and safety. **Conclusions:** This framework provides healthcare executives and program managers with evidence-based guidance for implementing and enhancing HITH programs, addressing a critical gap in governance and management literature.

## 1. Introduction

Hospital in the Home (HITH), also referred to as Hospital at Home (HaH) or Virtual Hospital (VH), has emerged as an innovative approach within the healthcare system in recent years. This approach represents a significant shift in caregiving to patients as it delivers hospital-quality treatment at patients’ homes [1]. This evolving model represents a comprehensive approach to healthcare delivery [2]. As healthcare systems struggle with the issues of ageing populations and an increasing number of chronic illnesses, leading to increased demand on inpatient hospital beds, HITH programs have gained popularity worldwide [3]. In addition to providing an alternative to overcapacity hospitals, HITH is in line with a greater focus on patient-centered care by allowing patients to receive medical treatment in comfortable surroundings [4]. HITH has become an attractive alternative to traditional inpatient services as healthcare professionals and authorities seek methods to improve care quality while controlling in hospital access, flow and costs. Recent evidence continues to demonstrate the emergence of HITH as a high-value model of care delivery [5].

HITH programs offer multiple benefits, including reduced hospital readmissions, lower healthcare costs, improved patient outcomes, and enhanced patient satisfaction through personalized care delivered in familiar home environments [6]. This alteration provides a more comfortable recovery in a familiar environment and minimizes the pressure on hospital resources in addition to possibly lowering the risk of hospital-acquired health issues [7]. Additionally, HITH initiatives have demonstrated promise in maintaining or improving patient outcomes and experience while reducing medical costs [8]. But fulfilment of these benefits requires efficient governance and management structures designed to meet certain challenges of delivering hospital-level care in different home environments. Understanding technology adoption is critical, with research identifying key drivers that influence stakeholder acceptance of new systems [9,10,11]. Selecting appropriate technology solutions, and making the right governance decisions significantly impact success and outcomes of technological innovations [12,13]. These insights directly apply to Hospital-in-the-Home programs, where adoption drivers, technology selection, and governance decisions determine program success.

Despite these promising benefits, however, healthcare organisations experience difficult management and implementation issues. Clinical procedures, integration of technology, and management of care need to be modified for HITH in comparison to standard medical facilities [1]. The complicated nature of the situation is increased by the requirement to maintain consistent standards of care quality, patient safety, and regulatory compliance in decentralized health care systems. Interestingly, even though HITH initiatives’ clinical results and cost-effectiveness have been the focus of many studies, there is relatively limited research on the governance models and the lessons learnt from management frameworks that are exclusive to these programs [14]. While HITH offers substantial benefits, comprehensive reviews highlight both the advantages and challenges of implementation [15].

To address this gap, HITH program managers and healthcare executives need clear guidelines on how to manage, develop, and execute these new healthcare approaches. To do so, this study aims to investigate and analyze the body of literature on HITH governance and management through a systematic review of published research and case studies to answer the following research question:

*RQ: “What lessons can be learnt from the governance and management of HITH initiatives?”*

This systematic review addresses a critical gap in the literature by providing the first comprehensive synthesis of governance and management lessons specifically for HITH programs. While previous systematic reviews [1,3,6,7,8] have focused primarily on clinical effectiveness and safety outcomes dating from early HITH implementations [4] to contemporary models, our study uniquely consolidates strategic and operational insights for healthcare executives and program managers.

## 2. Methodology

**Figure 1:**
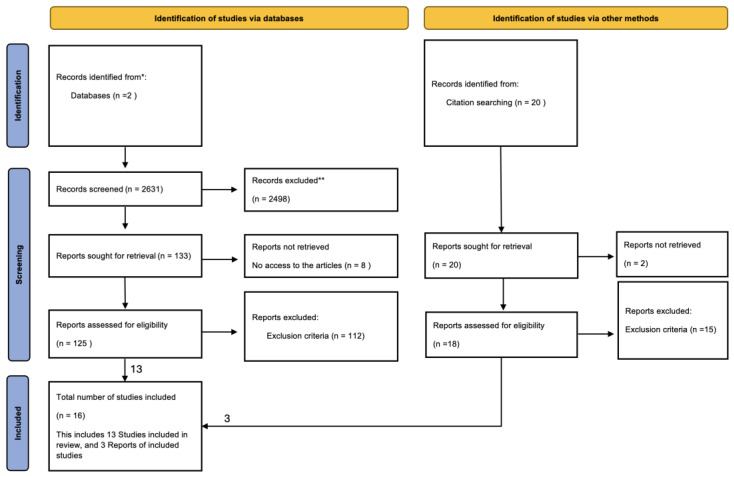
PRISMA 2020 flow diagram for systematic review of HITH governance and management lessons [7]. * In the identification phase, n = 2 indicates 2 databases searched (ScienceDirect and Scopus), yielding 2631 total records. ** Records were excluded based on the following criteria: not focused on HITH/hospital-at-home programs, not reporting governance or management lessons, editorials or commentaries without primary data, and duplicate publications. The notation n = 20 in the adjacent box indicates records identified from other sources (citation searching, reference lists). The review included 16 unique, independent studies (13 from database searching, 3 from other sources), with no duplicate reports of the same studies. In the flow diagram, n indicates the number of records/studies at each stage.

This study employed a systematic literature review approach to investigate the lessons learned in the governance and management of Hospital in the Home (HITH) initiatives. To ensure methodological accuracy and transparency, the review procedure followed the Preferred Reporting Items for Systematic Reviews and Meta-Analyses (PRISMA) guidelines which are a set of criteria based on evidence and intended to raise the quality and transparency of meta-analyses and systematic reviews [16]. The protocol for this systematic review was not prospectively registered in PROSPERO or other registries, as the focus on governance and management lessons (rather than clinical effectiveness outcomes) was determined to fall outside the typical scope of clinical trial registries. The study selection procedure is detailed in the PRISMA flow diagram presented in the Results section (Figure 1).

ScienceDirect and Scopus were selected as search databases due to their comprehensive coverage of healthcare management, nursing, and health services research literature. Scopus was specifically chosen for its superior multidisciplinary coverage (27,000+ indexed journals) including substantial overlap with PubMed/MEDLINE while offering stronger representation of management, organizational studies, and health services research literature—domains critical for identifying governance and management lessons. Unlike systematic reviews focused on clinical effectiveness (where MEDLINE is essential), our review targets organizational and strategic insights that span implementation science, health policy, and management literature where Scopus provides comprehensive coverage. ScienceDirect complemented this strategy by providing full-text access to nursing and healthcare management journals from major publishers, enabling thorough review of governance-related content often detailed in discussion sections rather than abstracts. This database selection was supplemented by citation searching and reference list examination to ensure comprehensive literature capture.

The search strategy employed free text terms rather than controlled vocabulary (e.g., MeSH terms) because: (1) the focus on governance and management lessons spans multidisciplinary literature beyond medical databases requiring MeSH; (2) HITH terminology varies internationally without standardized descriptors; and (3) both databases searched do not utilize MeSH indexing. The titles, abstracts, and author-supplied keywords of articles were searched using the following free text terms: “hospital in the home” OR “HITH” OR “hospital at home” OR “HaH” OR “virtual care”, AND “lesson” OR “lessons”, AND “management” OR “governance”. Boolean operators (OR, AND) were used to combine terms as indicated.

The database searches were conducted in December 2023 and yielded the following results: Scopus (n = 1847 records) and ScienceDirect (n = 784 records), totaling 2631 records before duplicate removal. After removing 412 duplicates, 2219 unique records underwent title and abstract screening. An additional 20 records were identified through citation searching and reference list examination of included studies and relevant systematic reviews.

The study selection process occurred in three stages following PRISMA guidelines. **Stage 1 (Title and Abstract Screening):** After conducting searches in two databases, all 2219 unique records were imported to Covidence (a systematic review management software). Two researchers (AP and NAE) independently screened titles and abstracts against inclusion criteria. Discrepancies were identified by the software and resolved through discussion, with 52 records advancing to full-text review. **Stage 2 (Full-Text Review):** The same two researchers independently assessed full-text articles for eligibility, with disagreements resolved through discussion and, when necessary, arbitration by a senior researcher (AHG). Thirteen studies from database searches plus three additional studies identified through citation searching met all inclusion criteria. **Stage 3 (Data Extraction):** A total of 16 papers were included for data extraction and analysis.

While data extraction was conducted independently by two researchers as described in Section 2.3, the initial thematic coding was conducted primarily by one investigator (AP) with systematic verification and arbitration by a senior investigator (AHG) rather than fully independent dual coding. Relevant outcomes were extracted from the reported findings, discussions, and tables in the reviewed studies through content analysis. The results from the included papers were organized into groups, analyzed based on recurring themes, and then summarized in a narrative format. While ScienceDirect offers extensive access to nursing and healthcare journals relevant to HITH programs. Having established our database selection strategy, we next defined explicit criteria to guide study selection.

### 2.1. Inclusion and Exclusion Criteria

Studies were included if they: (1) focused on Hospital in the Home, Hospital at Home, or Virtual Hospital programs; (2) reported lessons learned, best practices, governance structures, or management strategies; (3) were published in English in peer-reviewed journals; and (4) provided sufficient detail on program implementation or outcomes. Studies were excluded if they: (1) focused solely on clinical effectiveness without discussing organizational or governance aspects; (2) were conference abstracts, editorials, or commentaries without empirical data; or (3) examined only emergency department or outpatient virtual care without home-based hospitalization components.

To ensure methodological rigor and transparency, all data extraction forms, coding frameworks, and classification decisions were documented and are available upon request. The qualitative analysis process was iterative, with regular team meetings to discuss emerging themes and ensure consistency in lesson extraction and classification approaches.

### 2.2. Data Extraction and Qualitative Analysis

Data extraction was conducted independently by two researchers (AP and NAE) using a standardized form capturing study characteristics, HITH program details, and reported lessons or recommendations. Lessons were defined as actionable insights, best practices, or critical success factors explicitly stated by authors or derived from reported findings.

The analysis followed Braun and Clarke’s thematic analysis framework [17] through three stages: (1) initial open coding of all extracted lessons by two researchers independently; (2) axial coding to group related lessons into categories based on operational domains; and (3) selective coding to develop overarching themes. The coding was conducted using NVivo 14 qualitative data analysis software, which facilitated systematic organization and comparison of codes across researchers. Each researcher independently coded all 42 lessons, assigning them to operational categories. Discrepancies in coding were resolved through discussion between researchers, with a senior researcher (AHG) providing final arbitration when consensus could not be reached. The reliability of coding was established through calculation of Cohen’s kappa coefficient using a 2 × 2 contingency table comparing independent coding assignments for each lesson. This analysis achieved substantial agreement (Cohen’s kappa = 0.74) during the initial coding phase before proceeding with final classification and theme development.

### 2.3. Quality Assessment

The methodological quality of included studies was assessed using the Mixed Methods Appraisal Tool (MMAT) version 2018 [18], which is appropriate for systematic reviews including diverse study designs (quantitative, qualitative, and mixed methods studies). Two reviewers independently assessed each study using the relevant MMAT criteria based on study design. The MMAT evaluates five criteria relevant to each study type, with responses of ‘Yes,’ ‘No,’ or ‘Can’t tell’ for each criterion. For quantitative descriptive studies (program evaluations), we assessed sampling strategy, sample representativeness, appropriateness of measurements, risk of non-response bias, and statistical analysis appropriateness. For qualitative studies, we evaluated research approach appropriateness, data collection adequacy, findings substantiation, coherence of interpretation, and consideration of researchers’ influence. For mixed methods studies, we applied both quantitative and qualitative criteria plus integration assessment. Studies were classified as high quality (meeting 4–5 criteria), moderate quality (meeting 2–3 criteria), or low quality (meeting 0–1 criteria). Disagreements in quality ratings were resolved through discussion, with involvement of a senior reviewer when necessary. Quality scores were used to contextualize the strength of evidence supporting each lesson learned rather than for exclusion purposes, given the limited body of literature in this specialized field. Studies with higher methodological quality were given greater weight in the synthesis process, particularly when conflicting evidence was present.

## 3. Results

Figure 1 presents the systematic study selection process. The database searches yielded 2631 records, with an additional 20 records identified through citation searching and reference list examination. After removing duplicates and screening, 16 unique, independent studies met the inclusion criteria and were included in the qualitative synthesis. Of these 16 studies, 13 were identified through database searching (ScienceDirect and Scopus) and 3 were identified through other sources (citation searching and reference lists). Each of the 16 included studies represents a distinct HITH program or implementation; there are no multiple reports or secondary publications of the same study.

The quality assessment revealed that 12 studies (75%) met high methodological quality standards, 3 studies (19%) demonstrated moderate quality, and 1 study (6%) showed lower quality based on MMAT criteria. Higher quality studies primarily included well-designed program evaluations with clear methodology, appropriate data collection methods, and comprehensive outcome reporting. The lessons derived from higher quality studies showed greater consistency and provided stronger evidence for implementation recommendations.

**Figure 2:**
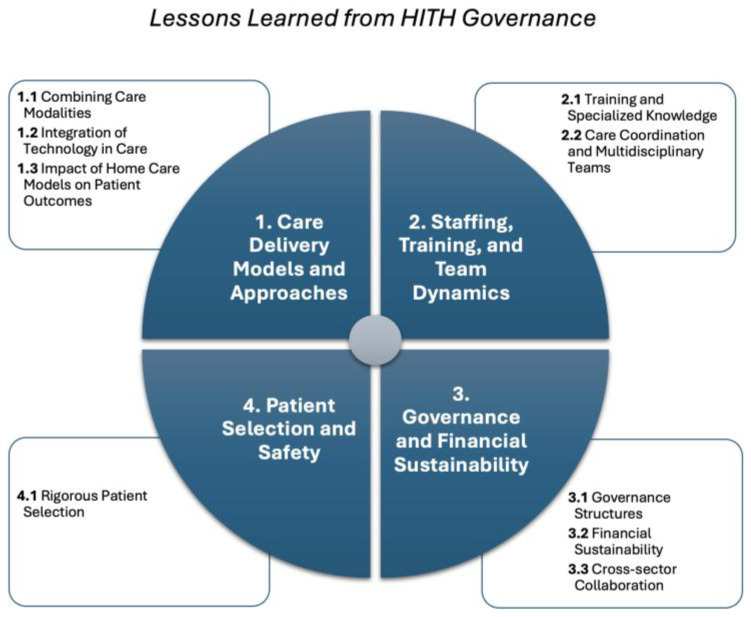
A framework presenting themes and categories for lessons learnt from managing HITH.

Building upon this quality-assured evidence base, the qualitative analysis yielded 42 lessons learned associated with managing different HITH programs, extracted from the 16 papers. These lessons learned are classified into nine categories including: combining care modalities, integration of technology in care, impact of home care models on patient outcomes, training and specialized knowledge, care coordination and multidisciplinary teams, governance structures, financial sustainability, cross-sector collaboration, and rigorous patient selection. These nine categories fall under four main themes including: care delivery models and approaches; staffing, training, and team dynamics; governance and financial sustainability; and patient selection and safety. Each lesson learned is represented by a code, where the first digit indicates the related theme, and the second digit indicates the group number to which each lesson belongs. Figure 2 shows the framework of lessons produced by this study. Figure 2 presents the comprehensive framework illustrating the hierarchical relationship between the four main themes, nine categories, and 42 individual lessons learned. This visual representation demonstrates how specific operational insights are systematically organized into broader strategic domains for HITH program governance and management. 

### Characteristics of Included Studies

Table 1 presents the characteristics of the 16 studies included in this systematic review, including study design, geographic location, HITH program type, and primary focus areas.

The four themes represent a hierarchical governance framework, progressing from operational foundations to strategic implementation. Themes 1 and 4 establish the operational parameters—defining *what* services HITH programs deliver and to *whom*. Theme 2 addresses the human resource infrastructure—*who* delivers these services and *how* they are prepared. Theme 3 integrates these operational and workforce elements within governance and financial structures that determine program sustainability and scalability. This framework recognizes that successful HITH governance requires alignment across all four dimensions, with lessons from operational delivery informing governance decisions, and governance structures enabling operational excellence.

The qualitative analysis process resulted in the extraction of 42 distinct lessons learned from the 16 included studies. Of these, 28 lessons (67%) were explicitly stated as recommendations or lessons by the original authors, while 14 lessons (33%) were derived through synthesis and interpretation of reported findings, outcomes, and successful program elements described in the studies. All interpreted lessons were grounded in specific data, cases, or outcomes reported in the original sources and underwent validation through the structured coding process described in the methodology.

It should be noted that while some categories may appear to have overlapping elements (particularly technology integration and team coordination), each category represents distinct operational domains: technology integration focuses on digital tools and platforms, while team coordination emphasizes interprofessional collaboration processes and human resource management. Table 2 indicated the characteristics of included studies.

The 42 lessons identified vary in their scope of applicability across different HITH program contexts. While the majority of lessons (approximately 67%) are generalizable across all HITH program types—including fundamental governance structures (Theme 3), basic technology integration principles (Category 1.2), and core staffing requirements (Category 2.1)—some lessons are more specialized for specific clinical areas. For example, lesson 4.1.5 regarding oncology toxicities provides valuable insights for programs serving similar high-acuity populations, demonstrating how core HITH principles can be adapted for specialized care scenarios. These condition-specific lessons, while representing approximately 24% of our findings, offer important guidance for program managers developing HITH services for specialized patient populations.

As indicated in Table 2, the first theme includes three groups of combining care modalities, integration of technology in care, and the impact of home care models on patient outcomes. Altogether, 16 lessons fall under the first theme. The first group includes four lessons learned related to the importance of integrating various forms of care such as virtual and in person care, as well as hybrid models in at-home programs. For instance, 1.1.3. emphasizes that the admission process is easier and less urgent for patients transitioning from an inpatient bed compared to those coming from the emergency department or clinic. This lesson was learned from the program for community-acquired pneumonia, congestive heart failure, chronic obstructive pulmonary disease, and cellulitis by Mader et al. [20]. 1.1.4. indicates the advantages of a hybrid telehealth physiotherapy model in a paediatric at-home setting and suggests increasing staff flexibility to schedule physiotherapy sessions for patients [21].

Extending beyond care modality combinations, the second group with eight lessons focuses on the integration of technology in HITH programs and brings together key lessons learned about the role of digital technologies in HITH programs. For instance, 1.2.1. illustrates how digital technologies allow physicians to extend their reach, which is especially valuable in rural areas and the area with less accessibility [22]. 1.2.2. emphasizes how remote monitoring and consultations can reduce geographical barriers and clinical uncertainty and can improve patient care [23]. The third group, with four lessons, highlights the impact of home care models on patient outcomes. For example, 1.3.2. demonstrates that the at-home model has been shown to reduce the length of stay compared to traditional hospital care, particularly in stroke rehabilitation, while also providing flexibility to adapt to different patient needs and health crises [26]. 1.3.4. reveals that shifting the focus from ‘readiness for discharge’ to ‘readiness for home care’ has improved transitions for dementia patients [27].

While Theme 1 establishes the care delivery models and technological infrastructure, Theme 2 addresses the critical human resource dimension required to operationalize these models. The second theme includes 10 lessons focusing on staffing, training, and team dynamics—recognizing that even the most sophisticated care models and technologies require appropriately trained, coordinated teams to achieve intended outcomes. The first group focuses on training and specialized knowledge, highlighting lessons learned from several HITH programs in effective care coordination. For instance, 2.1.1 shows the necessity of having a central point person to coordinate resources and triage patient calls in complex care scenarios [28]. According to Marshall et al. [14], centralizing training at regional hubs while managing routine follow-ups locally can enhance care quality, as noted in 2.1.4 in Table 2. According to the BTS Guidelines Development Group [29], in an at-home chronic obstructive pulmonary disease program, clinical responsibility and out-of-hours cover are suggested to be undertaken by the acute trust, and at the time of patients’ discharge, clinical responsibility should be formally transferred back to primary care. They also recommend that the home care team should be led by a specialist respiratory nurse, physiotherapist, or other suitably qualified health professional in a chronic obstructive pulmonary disease (2.1.6) [29].

The operational capabilities described in Themes 1 and 2—care delivery models and workforce competencies—must be embedded within sustainable organizational structures to achieve long-term viability. Theme 3 shifts focus from operational execution to strategic governance, encompassing 11 lessons across three critical dimensions: governance structures, financial sustainability, and cross-sector collaboration. Regarding governance, lesson 3.1.1 highlights the importance of having formalized governance structures in place to ensure transparency, accountability, and effective process management. Additionally, collaboration between management governance which focuses on business operations, and clinical governance is key to making patient-centered decisions [14]. Lesson 3.1.3 emphasizes how a centralized command center can maintain consistent patient volumes and outcomes, which allow the model to work effectively across hospitals in different geographical areas [19,27].

The financial matters are another area of focus under the third theme. For instance, 3.2.1 indicates the challenge of balancing immediate costs with long-term savings when expanding home-based acute care services [28]. Lesson 3.2.3 notes that although early-discharge models may be easier to implement and result in higher patient throughput, hospital substitution models offer more substantial cost savings and help reduce iatrogenic events. This is particularly relevant for programs treating conditions like congestive heart failure, chronic obstructive pulmonary disease, and cellulitis [20]. Beyond internal financial sustainability, external partnerships also emerged as critical success factors. Lesson 3.3.1 highlights the benefits of cross-sector collaboration between hospitals, nursing homes, and community providers. Such a collaboration helps ensure continuity of care and strengthens long-term care models [31]. Building strong relationships with external clinical partners (3.3.3) is another key point in the success of HITH programs. Clearly defining expectations during the contracting phase and holding regular check-ins can help address any emerging issues, as noted by Titchener et al. [32] in the context of a cancer at home program. Fulton et al. [28] indicates that partnerships with community paramedicine in home-based acute care settings result in faster response times and expanded treatment options (3.3.2).

Effective governance and sustainable operations (Themes 1–3) ultimately serve the fundamental purpose of delivering safe, appropriate care to suitable patient populations. Theme 4 returns to operational considerations with a critical gatekeeping function: patient selection and safety. This theme includes five lessons that determine which patients can safely benefit from HITH services, directly influencing program outcomes, resource utilization, and the viability of governance structures described in Theme 3. Lesson 4.1.1 highlights effective patient selection can significantly reduce emergency readmissions and through ensuring that only those patients who are suited for HITH programs are admitted to [19]. Similarly, lesson 4.1.2 emphasizes the importance of using validated and explicit criteria for selecting patients, which helps managing risks and decreasing the overall length of stay in HITH programs [20]. Lesson 4.1.4 addresses the critical importance of comprehensive environmental assessment in patient selection. The suitability of the home environment extends beyond simple adequacy to encompass specific safety criteria that must be systematically evaluated before a patient can be safely admitted to HITH care. This includes assessment of physical safety features (adequate space, lighting, and accessibility for care delivery), availability of essential utilities (running water, heating/cooling systems, and reliable electricity for medical equipment), infection control capability (appropriate cleanliness and designated space for medical procedures), and emergency access (safe entry and exit routes for emergency personnel if needed). The primary studies emphasized that environmental safety assessment should be conducted as a formal pre-admission evaluation, often involving home visits by nursing staff or case managers, to identify and address any safety concerns before care begins. Lesson 4.1.5 demonstrates that oncology-related toxicities, such as pain, nausea, vomiting, neutropenic fever, and infections, can be managed effectively with acute-level care at home. The lesson also indicates the importance of having an escalation protocol in place to transfer patients to the emergency department or ambulatory care center if they cannot be stabilized at home [32]. Table 3 shows the summary of primary findings and extracted lessons from included studies.

## 4. Discussion

This systematic review addresses a critical gap by consolidating governance and management lessons from diverse HITH programs. The identified framework of four themes and nine categories provides healthcare managers with a structured approach to program implementation and improvement, distinguishing between broadly applicable principles and specialized applications. The lessons identified emphasize that technology integration (Theme 1, Category 1.2) serves as both an enabler and differentiator across HITH models, from basic telehealth applications to sophisticated centralized command centers. Organizations should view technology adoption as a scalable continuum rather than an all-or-nothing implementation.

In HITH programs, establishing a formalized governance structure is crucial for ensuring transparent accountability and maintaining clear connections across all key processes that influence patient outcomes [14]. Therefore, understanding key accountabilities, fostering collaboration between management governance and clinical governance, as well as defining clinical responsibilities in different stages in HITH programs, can positively impact the performance of these programs. There needs to be a central point person for triaging and managing team resources to ensure effective operations [28]. The governance of HITH programs can also benefit from having a good relationship with external partners [32], and having collaboration among hospitals, nursing homes, and community providers [31]. According to Fulton et al. [28], partnership with community paramedicine allows for faster response times and provides more treatment tools in a HITH program. According to Titchener et al. [32], establishing a solid relationship with external partners is beneficial if expectations between the parties are clearly defined. According to Lim et al. [23], actively reviewing patients in hospital wards and maintaining regular communication with ward clinicians helps identify the right candidates for the HITH program.

The interconnections among themes reveal important strategic implications for HITH governance. For instance, patient selection criteria (Theme 4) directly impact staffing requirements and training needs (Theme 2), which in turn influence financial sustainability models (Theme 3, Category 3.2). Similarly, technology integration decisions (Theme 1, Category 1.2) necessitate corresponding investments in staff training and technical competencies (Theme 2), while governance structures (Theme 3, Category 3.1) must provide oversight mechanisms that ensure technology adoption aligns with patient safety protocols (Theme 4). Organizations should therefore approach HITH implementation holistically rather than addressing themes in isolation, recognizing that decisions in one domain create cascading implications across others.

Healthcare organizations implementing HITH programs should recognize that lessons learned span a spectrum from broadly applicable governance principles to specialized clinical applications. The framework allows for flexible implementation where organizations can prioritize fundamental lessons as foundational elements while selectively incorporating specialized lessons based on their specific patient populations and clinical focus areas. This approach maintains core governance and management principles while accommodating the diverse nature of HITH program implementations across different healthcare contexts.

The framework’s flexibility becomes particularly important when considering contextual variations. The implementation of HITH programs in resource-limited settings presents unique challenges that require adaptive strategies based on our identified lessons. For instance, while lesson 1.2.1 demonstrates that digital technologies allow physicians to extend their reach in rural areas [22], the prerequisite technological infrastructure may be limited in underserved regions. In such contexts, lessons from Theme 2 (staffing, training, and team dynamics) become particularly critical—specifically lesson 2.1.4, which suggests centralizing training at regional hubs while managing routine follow-ups locally [14]. This approach could be adapted for resource-constrained settings by establishing mobile training units or utilizing existing community health centers as training hubs. Additionally, lesson 3.3.2 regarding partnerships with community paramedicine [28] offers a practical solution for areas lacking robust emergency medical services, as community health workers could fill similar roles in providing rapid response capabilities.

The applicability of the identified lessons learned must be considered within the context of varying healthcare systems and economic environments. Our analysis reveals that the majority of included studies were conducted in high-income countries with well-established healthcare infrastructure, which may limit direct transferability to resource-constrained settings. In developed healthcare systems, technology-intensive solutions such as centralized command centers (Lesson 3.1.3) and advanced digital integration (Theme 1, Category 2) may be readily implementable, whereas resource-limited settings may need to prioritize foundational governance structures and cross-sector collaboration before advancing to complex technological solutions. Similarly, financial sustainability considerations differ markedly between systems-developed countries with established reimbursement mechanisms can focus on optimizing cost-effectiveness ratios, while developing nations may need to emphasize basic service delivery models that operate within constrained budgets and rely more heavily on community-based training approaches rather than centralized specialized training hubs.

The results of this research are based on a systematic literature review, without field data collection or expert opinions. Therefore, the validity threats and limitations present in our reviewed papers may also apply to this research. Although screening and data extraction were conducted independently by two researchers, the thematic coding process deviated from PRISMA’s recommended fully independent dual-reviewer approach. Initial coding was performed by one researcher with systematic verification by a senior researcher rather than independent parallel coding by two reviewers. While inter-rater reliability testing (Cohen’s kappa = 0.74) on a subset of lessons demonstrated substantial agreement, this approach may have introduced potential bias in theme development compared to fully independent dual coding throughout. In addition, systematic literature review studies are always limited by inclusion and exclusion criteria and the scope of available databases, which in this research can also be considered a sort of limitation. While we conducted quality assessment using established criteria, the diverse nature of study designs in HITH governance research (ranging from program evaluations to case studies) presented challenges in applying uniform quality standards. The limited body of high-quality experimental studies in this field necessitated inclusion of descriptive studies, which may limit the strength of some evidence supporting our lessons learned. Some primary studies might also have been overlooked because of the different terminology used by health services to describe HITH.

The use of two databases (Scopus and ScienceDirect) rather than a broader database search including MEDLINE, CINAHL, and Web of Science represents a limitation, though we believe this was mitigated by several factors: Scopus’s extensive journal coverage with substantial overlap with other databases, the multidisciplinary nature of governance research extending beyond clinically indexed literature, and supplementary citation searching that identified 19% of included studies. Future systematic reviews in this domain may benefit from incorporating additional databases to ensure maximum comprehensiveness, particularly CINAHL for nursing-specific governance literature and Web of Science for citation network analysis.

## 5. Conclusions

This systematic review provides the first comprehensive synthesis of governance and management lessons specifically for Hospital in the Home (HITH) programs, identifying 42 actionable lessons organized within a hierarchical framework of four themes and nine operational categories. Unlike previous reviews focused on clinical effectiveness, this study addresses a critical gap by providing healthcare managers and policymakers with evidence-based guidance for the organizational dimensions of HITH implementation.

### 5.1. Practical Implications for Healthcare Organizations

Healthcare organizations implementing or expanding HITH programs should adopt a phased approach guided by the four-theme framework. **First**, establish foundational governance structures with clear accountability mechanisms and cross-sector partnerships (Theme 3) before advancing to complex operational innovations. **Second**, invest in workforce development through regional training hubs and multidisciplinary coordination systems (Theme 2), recognizing that technology-enabled care models (Theme 1) require corresponding staff competencies. **Third**, implement rigorous patient selection protocols (Theme 4) from program inception, as appropriate patient targeting directly influences all other dimensions of program success. Organizations in resource-limited settings should prioritize foundational governance and training lessons before pursuing technology-intensive solutions, adapting digital innovations such as remote monitoring [22,23] to local infrastructure constraints while leveraging scalable training models [14].

### 5.2. Research Directions

Future research should address three critical evidence gaps. First, longitudinal cost-effectiveness studies examining sustained financial impacts over multiple years would support investment decisions currently based on short-term outcomes. Second, development of home-specific safety risk assessment models would address the unique considerations of delivering acute care outside traditional hospital settings. Third, investigation of technology adaptation strategies and scalable training models [14,22,23] for resource-constrained contexts would enable equitable HITH expansion across diverse healthcare systems. Future systematic reviews should consider broader database coverage including MEDLINE, CINAHL, and Web of Science to ensure maximum capture of nursing-specific and clinical implementation literature, particularly as HITH governance research continues to evolve and expand across disciplines. Additionally, while this systematic review provides a comprehensive framework of 42 lessons learned across nine categories, future work should develop detailed implementation guides with specific criteria, assessment tools, and contextual adaptation frameworks for each lesson. Such operational guidance would support healthcare organizations in translating these evidence-based lessons into concrete policies, procedures, and practices tailored to their specific organizational contexts and patient populations.

The framework presented in this review equips healthcare leaders with structured guidance to navigate HITH complexity, translating lessons from established programs into actionable strategies for implementation, optimization, and sustainable scale.

## Figures and Tables

**Table 1:** Characteristics of included studies.

Study	Country	Study Design	HITH Program Type	Primary Focus Area
Paulson et al. (2023) [19]	USA	Descriptive cohort	Hybrid virtual/in-person acute care	Patient selection, governance structure
Mader et al. (2008) [20]	USA	Program evaluation	Hospital substitution (CAP, CHF, COPD, cellulitis)	Care delivery model, financial sustainability
Benz et al. (2023) [21]	Australia	Case study	Pediatric telehealth physiotherapy	Technology integration, hybrid care model
Nair et al. (2023) [22]	USA	Implementation study	Virtual visit track for ED patients	Technology integration, care coordination
Lim et al. (2024) [23]	Singapore	Implementation study	General acute HITH	Patient selection, remote monitoring
Whitehead & Conley (2023) [24]	USA	Review/commentary	Remote patient monitoring in HITH	Technology integration, patient outcomes
Lopez et al. (2021) [25]	Canada	Program evaluation	Virtual cancer rehabilitation	Technology adaptation, pandemic response
Inzitari et al. (2021) [26]	Spain	Observational study	Geriatric HITH during COVID-19	Care model adaptation, patient outcomes
Toles et al. (2023) [27]	USA	Implementation study	Transitional care for dementia patients	Care coordination, patient-centered approaches
Fulton et al. (2022) [28]	USA	Program description	Comprehensive in-home care for complex needs	Governance, financial sustainability, partnerships
Marshall et al. (2015) [14]	New Zealand	Program evaluation	Home hemodialysis	Governance structure, training, infrastructure
BTS Guidelines (2007) [29]	UK	Clinical guideline	COPD hospital-at-home	Clinical responsibility, team leadership
Franzosa et al. (2021) [30]	USA	Qualitative study	Home-based primary care during COVID-19	Pandemic adaptation, care delivery
Wong et al. (2022) [31]	Canada	Program description	Collaborative nursing home care	Cross-sector collaboration, pandemic response
Titchener et al. (2021) [32]	USA	Program description	Oncology hospital-at-home	Patient selection, external partnerships
Lightwood et al. (1957) [33]	UK	Historical trial	Pediatric home care	Early home care model, outcomes

Note: CAP = Community-acquired pneumonia; CHF = Congestive heart failure; COPD = Chronic obstructive pulmonary disease; ED = Emergency department; HITH = Hospital in the Home.

**Table 2:** Lessons learned for managing and governing different HITH initiatives.

Theme	Category	Lessons Learned *	Reference
1. Care Delivery Models and Approaches	1. Combining care modalities	1.1.1. Combining virtual physicians with bedside nurses and vendor-managed supply chains can deliver acute care outside traditional hospital settings.	Paulson et al. [19]
		1.1.2. Combining virtual and in-person care increased patient volume, lowered costs, and enhanced scalability by using external vendors.	[19]
		1.1.3. The admission process is easier and less urgent for patients transitioning from an inpatient bed compared to those coming from the emergency department or clinic. Mader et al. [9] demonstrated this in their Veterans Affairs program treating community-acquired pneumonia, congestive heart failure, COPD, and cellulitis, where inpatient transfers showed smoother care transitions.	[20]
		1.1.4. Employing a hybrid telehealth physiotherapy model increased staff flexibility to accommodate physiotherapy sessions before and after school, as additional travel time during these peak traffic periods makes home visits inaccessible.	[21]
	2. Integration of technology in care	1.2.1. Digital technologies allowed physicians to extend their reach, especially valuable in rural areas.	[22]
		1.2.2. Leveraging telemedicine technologies, like remote monitoring and consultations, reduces geographical barriers and clinical uncertainty.	[23]
		1.2.3. Remote Patient Monitoring enhances economies of scale, reduces costs by expanding patient eligibility, and should be closely monitored for cost efficiency.	[24]
		1.2.4. Virtual care faced limitations in physical assessments like musculoskeletal and neurologic exams, leading to in-person waitlists.	[25]
		1.2.5. Telehealth hybrid models for paediatric physiotherapy showed no significant increase in adverse events and high acceptability.	[21]
		1.2.6. Video-based appointments provide a greater sense of confidence with the examination and care plan than telephone visits.	[25]
		1.2.7. Using vital sign remote patient monitoring in the HITH model could align with standard practice of checking vital signs every 4-8 h, allowing for safe care of moderate-acuity patients who usually aren’t included in HITH. Whitehead and Conley [13] specifically identified this as a mechanism to expand patient eligibility while maintaining safety standards	[24]
		1.2.8. Having a fully integrated electronic medical record system that connects inpatient, outpatient, and home care data can significantly enhance care coordination and information sharing among all providers.	[20]
	3. Impact of home care models on patient outcomes	1.3.1. The HITH model can lower the need for emergency readmissions by offering post-discharge restorative care.	[19]
		1.3.2. The HITH model has been shown to reduce the length of stay compared to traditional hospital care, particularly in stroke rehabilitation, and provides flexibility to adapt to different patient needs and health crises.	[26]
		1.3.3. Pre-discharge planning combined with telephone-based support reduced readmissions, supporting dementia caregivers.	[27]
		1.3.4. Shifting focus from “readiness for discharge” to “readiness for home care” helped improve transitions for dementia patients.	[27]
2. Staffing, Training, and Team Dynamics	1. Training and specialized knowledge	2.1.1. A central point person is necessary for coordinating resources and triaging patient calls in complex care scenarios.	[28]
		2.1.2. Staffing for home-based care should prioritize providers with experience in caring for medically vulnerable populations.	[28]
		2.1.3. Clinicians experienced in home-based care are more likely to identify suitable patients compared to hospital ward clinicians.	[23]
		2.1.4. Centralizing training at regional hubs while managing routine follow-ups locally can enhance care quality.	[14]
		2.1.5. The lead clinician should be a consultant respiratory physician, supported by trainee junior medical staff.	[29]
		2.1.6. The home care team should be led by a specialist respiratory nurse, physiotherapist, or appropriately qualified health professional.	[29]
	2. Care coordination and multidisciplinary teams	2.2.1. Daily “huddles” and case reviews keep multidisciplinary teams engaged and responsive to patient needs.	[30]
		2.2.2. Proactive review of patients in hospital wards and regular communication with clinicians facilitated appropriate referrals for home-based care. This approach helps identify the right candidates for home-based treatments effectively.	[23]
		2.2.3. Having a fully integrated electronic medical record system that connects inpatient, outpatient, and home care data can significantly enhance care coordination and information sharing among all providers.	[20]
		2.2.4. Using an in-house home care team familiar with patient needs and experienced in collaborating with physicians can lead to better care coordination and lower complication rates.	[20]
3. Governance and Financial Sustainability	1. Governance structures	3.1.1. Formalized governance structures ensure transparent accountability and effective process management.	[14]
		3.1.2. Collaboration between management governance (focused on business compliance) and clinical governance (focused on patient care) is essential for ensuring patient-centred decisions.	[14]
		3.1.3. A centralized command centre allowed for consistent patient volumes and outcomes across geographically separate regions. Paulson et al. [8] demonstrated this in their hybrid hospital-at-home model implemented across two different geographical regions using a single command centre, achieving consistent outcomes in both locations.	[19]
		3.1.4. After recruitment to HITH, clinical responsibility and out-of-hours cover should be undertaken by the acute trust.	[29]
		3.1.5. When the patient is discharged from HITH program, clinical responsibility should be formally transferred back to primary care either by fax or by email.	[29]
	2. Financial sustainability	3.2.1. Balancing long-term savings with the immediate costs of expanding home-based acute care services was challenging, requiring financial planning.	[28]
		3.2.2. Securing reimbursement for services like nurse practitioner visits helped mitigate cash flow challenges.	[28]
		3.2.3. Early-discharge models might be easier to develop and implement and provide higher patient throughput. However, hospital substitution models offer more significant cost savings and reduced iatrogenic events.	[20]
	3. Cross-sector collaboration	3.3.1. Cross-sector collaboration between hospitals, nursing homes, and community providers fostered care continuity, enhancing long-term care models.	[31]
		3.3.2. Partnership with community paramedicine allows for faster response times and provides us with more treatment tools.	[28]
		3.3.3. Establishing a solid relationship with external clinical partners is crucial. Clearly define expectations during the contracting phase and conduct regular check-ins to resolve any emerging issues.	[32]
4. Patient Selection and Safety	1. Rigorous patient selection	4.1.1. Effective patient selection for home-based care reduces emergency readmissions.	[19]
		4.1.2. Using validated and explicit criteria for patient selection helps in managing patient risk, reducing complications, and potentially decreasing the overall length of stay at home.	[20]
		4.1.3. Rigorous patient selection and restorative care options enabled the program to achieve significantly better outcomes.	[19]
		4.1.4. Providing home care to a patient depends on factors such as the requirement for fixed hospital equipment, comprehensive environmental safety assessment of the living environment (including physical safety features, utility availability, infection control capability, and emergency access), and the readiness of involved parties (family members, the family doctor, and the hospital team leader) to take on additional responsibilities	[33]
		4.1.5. Oncology toxicities issues such as pain, nausea and vomiting, neutropenic fever, and infections can be effectively managed with acute-level care at home. An escalation protocol should employ to return patients to the emergency department or ambulatory care centre if they could not be stabilized at home.	[32]

* Some lessons represent synthesis of findings across multiple studies or interpretation of reported outcomes to derive actionable insights for HITH program management. All interpretations are grounded in specific data and cases reported in the original sources.

**Table 3:** Summary of primary findings and extracted lessons from included studies.

Study	HITH Program Context	Primary Findings Reported	Lessons Extracted (Code)
Paulson et al. (2023) [19]	Hybrid virtual/in-person acute care across two regions	Centralized command center maintained consistent outcomes across geographically separate hospitals; effective patient selection reduced ED readmissions	3.1.3, 4.1.1
Mader et al. (2008) [20]	Hospital substitution for CAP, CHF, COPD, cellulitis	Admission easier from inpatient beds vs. ED; substitution models offer greater cost savings than early discharge; validated patient criteria reduced LOS	1.1.3, 3.2.3, 4.1.2
Benz et al. (2023) [21]	Pediatric telehealth physiotherapy for CF	Hybrid model increased staff scheduling flexibility; reduced travel time during peak traffic	1.1.4, 1.2.4
Nair et al. (2023) [22]	Virtual ED fast track	Digital technologies extended physician reach, especially valuable in rural areas	1.2.1
Lim et al. (2024) [23]	General acute HITH	Active ward review and clinician communication improved patient identification; remote monitoring reduced geographical barriers	1.2.2, 4.1.3
Whitehead & Conley (2023) [24]	Remote patient monitoring overview	Technology integration enhances HITH outcomes and enables program expansion	1.2.3, 1.3.1
Lopez et al. (2021) [25]	Virtual cancer rehabilitation during COVID-19	Rapid technology adaptation maintained service continuity; virtual delivery achieved comparable outcomes	1.3.3, 2.2.2
Inzitari et al. (2021) [26]	Geriatric HITH during COVID-19	At-home model reduced LOS in stroke rehabilitation; flexibility to adapt to health crises	1.3.2
Toles et al. (2023) [27]	Transitional care for dementia patients	Shifting focus from ‘discharge readiness’ to ‘home readiness’ improved transitions; caregiver engagement critical	1.3.4, 2.2.4
Fulton et al. (2022) [28]	Comprehensive in-home care for complex needs	Central coordinator essential for resource management; partnership with paramedicine improved response times; balancing immediate costs with long-term savings challenging	2.1.1, 3.2.1, 3.3.2
Marshall et al. (2015) [14]	Home hemodialysis	Formalized governance ensured transparency and accountability; centralized training with local follow-up enhanced quality; management-clinical governance collaboration improved decisions	2.1.4, 3.1.1, 3.1.2
BTS Guidelines (2007) [29]	COPD hospital-at-home	Specialist nurse/physiotherapist leadership required; formal transfer of clinical responsibility at discharge; acute trust maintains out-of-hours responsibility	2.1.6, 2.2.1, 2.2.3
Franzosa et al. (2021) [30]	Home-based primary care during COVID-19	Pandemic accelerated technology adoption; interprofessional coordination essential for complex patients	2.1.2, 2.2.5
Wong et al. (2022) [31]	Collaborative nursing home care during COVID-19	Cross-sector collaboration (hospitals, nursing homes, community) ensured care continuity	3.3.1
Titchener et al. (2021) [32]	Oncology hospital-at-home	Clearly defined partner expectations critical; regular check-ins addressed emerging issues; oncology toxicities manageable at home with escalation protocols	3.3.3, 4.1.5
Lightwood et al. (1957) [33]	Pediatric home care trial	Early evidence of home care feasibility for pediatric populations	1.1.1, 1.1.2

Note: CAP = Community-acquired pneumonia; CF = Cystic fibrosis; CHF = Congestive heart failure; COPD = Chronic obstructive pulmonary disease; ED = Emergency department; LOS = Length of stay.

## Data Availability

No new data were created or analyzed in this study.

## Public Involvement Statement

No public involvement in any aspect of this research.

## Guidelines and Standards Statement

This manuscript was drafted in accordance with the PRISMA 2020 reporting guideline for systematic reviews and meta-analyses (Moher et al., 2009) [34].
